# Hospital preparedness for COVID-19 in Indonesia

**DOI:** 10.3389/fpubh.2023.1187698

**Published:** 2023-07-17

**Authors:** Fatma Lestari, Abdul Kadir, Attika Puspitasari, Suparni, Oktomi Wijaya, Herlina J. EL-Matury, Duta Liana, Riza Yosia Sunindijo, Achir Yani Hamid, Fira Azzahra

**Affiliations:** ^1^Occupational Health and Safety Department, Faculty of Health, Universitas Indonesia, Depok, Indonesia; ^2^Disaster Risk Reduction Center (DRRC) Universitas Indonesia, Depok, Indonesia; ^3^Department of Public Health, STIKes Dharma Husada, Bandung, Indonesia; ^4^Faculty of Public Health, Universitas Ahmad Dahlan, Yogyakarta, Indonesia; ^5^Faculty of Public Health, Institut Kesehatan Deli Husada Deli Tua, Medan, Indonesia; ^6^Health Administration and Policy Department, Faculty of Public Health, Universitas Indonesia, Depok, Indonesia; ^7^School of Built Environment, UNSW Sydney, Sydney, NSW, Australia; ^8^Faculty of Nursing, Universitas Indonesia, Depok, Indonesia; ^9^Faculty of Medicine, Universitas Indonesia, Jakarta, Indonesia

**Keywords:** hospital readiness checklist for COVID-19, hospital preparedness, hospital readiness, information system, Indonesia

## Abstract

**Introduction:**

As a disaster-prone country, hospital preparedness in dealing with disasters in Indonesia is essential. This research, therefore, focuses specifically on hospital preparedness for COVID-19 in Indonesia, which is important given the indication that the pandemic will last for the foreseeable future.

**Methods:**

During March to September 2022, a cross-sectional approach and a quantitative study was conducted in accordance with the research objective to assess hospital preparedness for the COVID-19 pandemic. This research shows the level of readiness based on the 12 components of the rapid hospital readiness checklist for COVID-19 published by the World Health Organization (WHO). Evaluators from 11 hospitals in four provinces in Indonesia (Capital Special Region of Jakarta, West Java, Special Region of Yogyakarta, and North Sumatra) filled out the form in the COVID-19 Hospital Preparedness Information system, which was developed to assess the level of hospital readiness.

**Results:**

The results show that hospitals in Capital Special Region of Jakarta and Special Region of Yogyakarta have adequate level (≥ 80%). Meanwhile, the readiness level of hospitals in West Java and North Sumatra varies from adequate level (≥ 80%), moderate level (50% – 79%), to not ready level (≤ 50%).

**Conclusion:**

The findings and the methods adopted in this research are valuable for policymakers and health professionals to have a holistic view of hospital preparedness for COVID-19 in Indonesia so that resources can be allocated more effectively to improve readiness.

## 1. Introduction

Indonesia, known for its Ring of Fire, is a high-risk area for various disasters, including the COVID-19 pandemic. Since its discovery in late 2019 and being declared a worldwide pandemic in March 2020, COVID-19’s effects have been holistic to almost every aspect of life. Since February 2020, Indonesia has detected more than six million positive cases with more than one hundred thousand deaths (1). It is, therefore, essential to evaluate and analyze the preparedness of healthcare facilities in Indonesia.

With the emergence of new variants, healthcare services’ condition has worsened. For instance, the Omicron variant caused another urgent public health alert due to its massive mutation and rising reinfections risk (2). New variants can have detrimental impacts on multiple sectors of healthcare facilities, particularly in the prevention and treatment aspects.

Hospitals should be able to function during and after the occurrence of disasters. As the frontline entity in handling COVID-19 cases, hospitals have essential roles in providing health services for the affected population. Hospital preparedness in facing disaster has been a concern for a long time. It has been included in Hyogo Framework for Action 2005–2015 (3) and Sendai Framework for Disaster Risk Reduction 2015–2030 (4), highlighting the essence of safe and effective operation during and after disaster events especially in COVID-19 referral hospitals. When a hospital considered an essential asset in disasters experiences faults or structural damages, it disrupts health services that directly affect the disaster-affected population’s safety and security. Therefore, it is crucial to study and analyze the level of preparedness of COVID-19 referral hospitals in Indonesia in facing the COVID-19 pandemic, which will likely last the foreseeable future.

To assess the preparedness of COVID-19 referral hospitals, WHO has published the rapid hospital readiness checklist for COVID-19 which includes 12 holistic components that hospitals should be aware of and maintain during the ongoing COVID-19 pandemic. Gaps within the 12 components of COVID-19 Hospital Preparedness can also be examined, resulting in improvements in the assessed hospitals.

## 2. Literature review

Previous studies conducted at pediatric and adult ICUs at Cairo University (5) and 22 Eastern Mediterranean Region (6) used the questionnaire based on the Hospital Readiness Checklist for COVID-19 developed by WHO Regional Office for the Eastern Mediterranean Region. It comprises 10 key components: leadership and coordination; operational support; logistics and supply management; information; communication; human resources; continuity of essential services and surge capacity; rapid identification; diagnosis; isolation and case management; and infection prevention and control (7). These studies found the intermediate level of hospital readiness at the initial outbreak of COVID-19 (5, 6).

Other studies have also used the Comprehensive Hospital Preparedness Checklist for COVID-19 published by the United States Centers for Disease Control and Prevention (CDC) (8, 9). This checklist consists of: the structure of planning and decision-making; development of a written COVID-19 plan; and elements of a COVID-19 plan (general, facility communications, consumable and durable medical equipment and supplies; identification and management of ill patients; visitors access and movement within the facility; occupational health; education and training; and healthcare services/surge capacity) (10). These studies concluded that hospitals need higher preparedness for COVID-19 services (8, 9).

Another study in Nigeria used the Hospital Readiness Checklist for COVID-19 released by WHO regional office for Europe (11). This checklist comprises the incident management system; surge capacity; infection prevention and control; case management; human resources; continuity of essential health services and patient care; surveillance: early warning and monitoring; communication; logistics and management of supplies, including pharmaceuticals; laboratory services; essential support services (12). This research adds two more components: staff welfare and availability of necessary items. This research concluded that most hospitals in Nigeria needed to be sufficiently prepared to deal with the COVID-19 outbreak (11).

Another previous study in Mazandaran Province, Iran, used a standard checklist of the Pan American World Health Organization (PAHO 2019) (13). The checklist comprises 10 components: incident management system; coordination; information management; logistics; finance and administration; detection; diagnosis; isolation; cases management; and prevention and infection control. The hospitals’ readiness to face the COVID-19 pandemic outbreak were at the good level (13).

The Director-general of Health Services of Indonesia also issued the Monitoring and Evaluation of Hospital Readiness during COVID-19 Pandemic Guidelines. The monitoring and evaluation which were done using instruments adopted from the Rapid Hospital Readiness Checklist published by World Health Organization (WHO), assessed hospitals’ readiness in governance, structure, plans, and hospital protocols in dealing with the COVID-19 pandemic (14). The checklist consists of 12 key components: leadership and incident management system; coordination and communication; surveillance and information management; risk communication and community engagement; administration, finance, and business continuity; human resources; surge capacity; continuity of essential support services; potential management; occupational health, mental health, and psychosocial support; rapid identification and diagnosis; and infection prevention and control (15).

The checklist is only available as an Excel spreadsheet and the supporting documents cannot be directly uploaded into the Excel files. Supporting documents must be submitted to District Health Office (DHO) and DHO will manually access, recapitulates, and maps hospital readiness in their respective areas. The recapitulation results are then forwarded to the Ministry of Health (16). Manually assessing checklists is time-consuming and there needs to be a feature for data saving and uploading supporting documents, which can result in lost or corrupted data (16).

Based on previous research, the COVID-19 Hospital Preparedness information system was created to assess hospital preparedness automatically. Evaluators are asked to fill out the form on this information system. This assessment form uses the 12 components from the Rapid Hospital Readiness Checklist for COVID-19 proposed by World Health Organization (WHO) (15). The checklist aims to help hospitals prepare for COVID-19 patient management by optimizing hospital capacities. The checklist takes into account a wide range of issues, including the need to provide continuing care to patients with acute or chronic illnesses; the laboratory services needed; hospitals information management; the need to train staff and other personnel; protection of healthcare workers, patients and visitors; and the needs for mental health and psychosocial support for all hospital staff (both medical and non-medical) (15). While completing the checklist, users should also consider the health system’s challenges in ensuring preparedness for other outbreaks and concurrent emergencies. The hospital score and achievement percentage are calculated automatically and displayed in tables and radar charts, making it easier for respondents to read the assessment results.

## 3. Materials and methods

This study was conducted using a cross-sectional approach and is a quantitative study in accordance with the research objective to assess hospital preparedness for the COVID-19 pandemic. Quantitative methods are characterized by collecting numerical data, using deductive reasoning, using natural science approaches to explain social phenomena, and having an objective conception of social reality (17). In the context of this study, numerical data was obtained by calculating the hospital safety index during the situation of COVID-19 pandemic.

### 3.1. Data sampling

Four provinces in Indonesia were selected as research locations: Central Special Region of Jakarta, West Java, Special Region of Yogyakarta, and North Sumatra. These locations were chosen because they have a high level of population mobility and a high number of confirmed COVID-19 cases. In addition, hospitals in Indonesia consist of Government Hospitals, Non-Governmental/Private Hospitals, the selected sample hospitals are these both hospital which are COVID-19 Patient Referral Hospitals according to the list of COVID-19 Referral Hospitals in Indonesia. All participating hospitals had received permission from the authority to participate in this study. The sampling technique used in this study is a non-probabilistic sampling technique based on the research objectives and the permission obtained from the research locations. Moreover, there are 11 hospitals selected: Capital Special Region of Jakarta (4 Hospitals), West Java (3 Hospitals), Special Region of Yogyakarta (2 Hospitals), and North Sumatra (2 Hospitals). The study was conducted from March to September 2022.

### 3.2. Data collection

Data were collected by the evaluator team, which consists of representatives from hospital management, technical division, administrative division, finance division, and medical team (doctors and nurses). The evaluators in this study were the authors who have background education and expertise in hospital disaster management. The level and value of each element was determined through the consensus of all evaluators. Evaluators already have the skills and competencies to assess hospital preparedness for disasters, and have conducted HSI assessments in several hospitals in Indonesia. Evaluators from 11 hospitals in four provinces (Capital Special Region of Jakarta, West Java, Special Region of Yogyakarta, and North Sumatra) filled out the form in the COVID-19 Hospital Preparedness information system. As stated earlier, this form uses the 12 components from the Rapid Hospital Readiness Checklist for COVID-19 developed by WHO (13) (Table 1). This instrument includes 12 Key Components and each key component has several Recommended Actions (sub-components) as follows:

**Table 1:** COVID-19 hospital preparedness checklist (15).

Key components	Recommended actions
1. Leadership and incident management system (15, 18)	The hospital has made emergency response plans for COVID-19, COVID-19 countermeasures team or task force and has been activated
	Areas or rooms for COVID-19 operation control centers have been determined and available to manage COVID-19 pandemic. The areas or rooms are equipped, safe, protected, and easily accessible by hospital staff to meet and coordinate
	Designated incident manager has been assigned to lead operation responses and improve hospital preparedness for COVID-19 risk management
	The assigned leader in the incident management team ensures appropriate inputs for decision-making, coordination, communication, and evidence-based management of COVID-19 pandemic
	Hospital has business continuity plans tested through COVID-19 pandemic simulation exercises
	The hospital has mechanisms to coordinate with the central and local governments and community for COVID-19 prevention, preparedness, response, and recovery
	All guidelines and documents related to COVID-19 risk management are available for all hospital staff
2. Coordination and communication (7, 12, 15, 19)	**Internal communication (within the hospital)**
	Communication plans and Standard Operating Procedures (SOP) for all hospital staff, patients, and visitors have been created and activated, including the duties and responsibilities of hospital staff and contact details (mobile number, email, and call sign)
	System and hospital communication system equipment for handling the COVID-19 pandemic are available and have been tested and function optimally in quality and quantity; the system can use telephones, cell phones, pagers, satellite phones, radio, and internet access. Backup communication systems are available
	All hospital staff, both medical and non-medical, have been briefed and trained regarding COVID-19 emergency policies and procedures to fulfill two-way communication with hospital management, staff, and visitors
	**External communication and coordination**
	The hospital’s COVID-19 incident management team or countermeasures task force has activated mechanisms to coordinate and communicate, e.g., with the Ministry of Health, National and Regional Agency for Disaster Management, to ensure that consistent approaches are taken in managing COVID-19
	Spokespersons for handling COVID-19 have been designated and trained
	All stakeholders involved in COVID-19 management, including media partners, are available
3. Surveillance and information management (15, 20)	**Surveillance**
	Hospital staff have been informed and trained regarding the definition of COVID-19 case, close contact, and quarantine system
	Standard forms are available to report COVID-19 case information to centralized health information system within 24 h of identification cases
	Standard Operating Procedures (SOP) for collecting, confirming, and validating COVID-19 data have been developed and are available to designated staff
	**Hospital information management**
	Hospital has designated staff to collect, analyze and disseminate data related to COVID-19 and services given by the hospital, followed by appropriate procedures
	Hospital has a system to ensure the system for documentation and safe storage location for COVID-19 information; backup system is available for now and in the future
	Mechanisms are available to collect feedback from patients and guests regarding COVID-19 management and operational mechanisms
4. Risk communication and community engagement (15, 21)	Protocols and Standard Operating Procedures (SOP) of risk communication regarding prevention and infection control are available for all staff, patients, guests, and other stakeholders, including community members
	Key messages for COVID-19 risk communication have been developed and updated based on situation developments and evidence-based technical guidelines
	Hospital staff who has been designated to update procedures and communication materials to manage COVID-19 issues and ensure everyone is kept informed about the COVID-19 pandemic
	Hospital staff are briefed regularly regarding COVID-19 risk communication messages and community engagement has been carried out
5. Administration, finance, and business continuity (15, 22)	All procedures for administration and finance are available for COVID-19 management, including purchasing, supplying, and required services
	Guidelines and administrative policies are available for hospital staff to deal with the COVID-19 pandemic
	Liability and insurance coverage and COVID-19 management procedures have been reviewed, including recruitment procedures and resource arrangements for temporary medical licenses
	Systems are available for fee waivers for COVID-19 health services (e.g., for COVID-19 testing and case management)
	Turnover and absenteeism of staff have been included in the hospital’s strategy to prevent staff burnout due to the COVID-19 workload and ensure the continuity of healthcare
	The incident management team has methods for assessing and identifying the expansion of inpatient, outpatient, and Intensive Care Unit (ICU) areas (including staff capacity and supply) if COVID-19 cases increase
	COVID-19 plans are available to refer or outsource non-critical services to appropriate alternative health facilities (e.g., home care services for people with minor illnesses that can be done by telemedicine)
	Hospital business continuity plans have been developed and tested to deal with the COVID-19 pandemic
6. Human resources (15, 23)	Hospital staff contact directory has been updated to use by the incident management team and manage hospital staff needs for COVID-19 management
	Hospital staff have been briefed, trained, and taken part in COVID-19 handling exercises in their work areas, including prevention, infection control, and clinical management, thereby ensuring the safety and competence of hospital staff
	Hospital administration has estimated the human resource capacity for preparedness and response to the spike potential of COVID-19 cases
	Hospital has identified the maximum number of staff (medical and non-medical) required to ensure the continuity of essential services during the COVID-19 pandemic
	Procedures to support new goals and reassign for hospital staff are available (e.g., teleworking available for staff with medical conditions at high risk for complications), with hospital strategy defining duties and responsibilities for managing COVID-19 risks
	Procedures for monitoring occupational health hazards are available to ensure the safety of hospital staff to mitigate the COVID-19 risk
7. Surge capacity (15, 24, 25)	The hospital has plans for capacity enhancement and rapid additions to address issues such as staffing, supply, logistics, and equipment; expertise for critical care area, and how to increase the number of beds based on real-time calculations; and there are also plans to increase all key activities in handling the increasing number of COVID-19 cases
	Hospital is part of the system or central surge mechanisms
	Procedures are available for COVID-19 management to improve supply chains for essential medicines, and diagnostics (including laboratory reagents, Personal Protective Equipment (PPE) and test kits; supply for clinical services, therapeutic measures, and clinical management)
	Hospital has agreements and Memorandum of Understanding (MoU) with the Ministry of Health or other institution to purchase equipment needed for capacity enhancement (such as mechanical ventilators, oxygen cylinders, *et cetera*)
	Human resource capacity enhancement available and updated, including names and contact details of volunteers (such as retirees, military health workers, medical students and senior nurses; community volunteers), with databases of backup staff
8. Continuity of essential support services (15, 26–28)	The hospital has identified and prioritized essential support services that must be available at all times and situations, with sufficient resources and backups to ensure the continuity of these services
	Hospital has identified the backup resources needed to keep essential support services functioning optimally, including human resources, finance, logistics, supply, beds, ICU, additional capacity rooms or areas for mortuary facilities, corpse bags, electricity, communications, water, and laundry services
	Hospital maintenance, supplies, and inventory systems are available for food, oxygen, cleaning materials, and disinfectants
	Hospital security system has identified potential safety hazards and health, including security access to the hospital, maintaining physical distance for a minimum of 1 meter; using masks if someone has symptoms of COVID-19, patient flow, traffic, parking and guest access; and stocks of essential pharmaceutical ingredients. The hospital also has mitigation plans for security risks
	Hospital has tested the expansion plan for clinical management (such as contingency plan to build additional isolation wards), and the waste management of the hospital has been integrated with the water, sanitation, and hygiene (WASH) system
	Hospital information management system available to monitor routine essential health service usage that is not related to COVID-19 through predetermined indicators
9. Patient management (15, 29–35)	Hospital updated protocols to provide essential care services for COVID-19 patients based on WHO guidelines; these guidelines are available and functional to all healthcare providers
	Procedures and provisions for receiving patients and transferring them to the areas or hospital isolation rooms are available, and other diagnostic and therapeutic support services are also available
	Hospital already has protocol for actions that have not been clinically tested/ are still trial / unregistered emergency measures, ethically approved clinical trials or Monitored Emergency Use of Unregistered Interventions (known as the MEURI framework) with close monitoring
	Hospital staff implements prevention protocol, infection control, and secure hospital networks and transport services before and after referral, including patients transferred from home care services
10. Occupational health, mental health, and psychosocial support (15, 36–39)	Hospital staff are protected, trained, and equipped with Personal Protective Equipment (PPE) in providing medical services to patients with suspected, probable or confirmed cases of COVID-19, including screening, resuscitation, initial stabilization, supportive therapy, and prevention of complications
	Hospital has the policy and capacity to manage occupational safety and health and prevent infection control from protecting hospital staff. Medical surveillance for hospital staff, suspected cases for their family, and close contacts; creating a blame-free environment and eliminating stigma against staff exposed to COVID-19
	Psychosocial and mental health support is available for hospital staff, family, and patients
	Standard Operating Procedures (SOP) for mental health screening are available for COVID-19 patients, their families, and hospital staff if emergency response increases
	All hospital staff have been trained in basic Occupational Safety and Health (OSH) and psychological first aid and know when to seek support services
11. Rapid identification and diagnosis (15, 31, 40)	Hospital staff have been trained accurately in rapid identification, and timely screening of suspected COVID-19 cases, by reporting to designated departments
	A communication and monitoring system allows for vigilance and reporting of suspected COVID-19 cases in all hospital areas, including entrances, reception areas, and patient reception
	Triage procedures are available in the emergency unit, focusing on rapid identification, isolation, and testing of patients with acute respiratory infection symptoms
	Hospital staff have been trained regarding standard procedures for taking samples and sending them to referral laboratories according to the latest recommendations and procedures for laboratory referrals
	If the hospital has the capacity for its testing laboratory, it has adopted the standard system for COVID-19 testing, supported by the availability of reagents and test kits
	Information and posters regarding Personal Protective Equipment (PPE) and biosafety have been displayed in laboratories and reception areas to safely handle samples, including their disposal
12. Infection prevention and control (15, 27, 32, 41–47)	Prevention Protocols and infection control with standard procedures for managing COVID-19 are available and functional, and all hospital staff have been trained on these protocols, including mechanisms for periodic monitoring
	Personal Protective Equipment (PPE) are available in sufficient quantity (such as medical masks, N95 masks or FFP2 respirators; gloves, gowns, and eye protection) and easily accessible to all staff designated to treat COVID-19 patients
	Hospital staff have been trained to identify and screen suspected COVID-19 cases, including screening for all patients, guests, and hospital staff
	Isolation areas are available for suspected, probable, and confirmed cases of COVID-19 with clear instructions or signs, adequate equipment, and ventilation
	Vigilance for airborne transmission using negative pressure room, minimum 12 air changes/h, and control of airflow direction when using mechanical ventilation. (negative pressure is mandatory to prevent cross-contamination from one room to another)
	Standards and vigilance of prevention and infection control are applied for case management in admission and referral persons with suspected, probable, and COVID-19 confirmed cases
	Hospital staff use airborne precautions during aerosol-generating medical procedures such as tracheal intubation, tracheotomy, cardiopulmonary resuscitation, bronchoscopy, and autopsy
	Precautions are available at the entrance areas and all areas of the hospital (hand washing facilities are equipped with water, soap, tissue, hand sanitizer, and rubbish bin at strategic locations in hospital areas)
	Posters regarding hygiene and cleanliness are available in the appropriate language and clear illustrations in various hospital areas, including hand washing information, cough etiquette, and social distancing
	Protocols are available to avoid transporting COVID-19 patients out of their rooms
	Hospital staff have been trained according to WHO guidelines regarding technical infection prevention and control, especially hand washing, respiratory hygiene, cough etiquette, social distancing, and using Personal Protective Equipment (PPE)
	Policies are available and implemented to ensure that beds are spaced at least 1 meter apart, regardless of suspected COVID-19
	All surfaces in hospitals and ambulances are regularly cleaned and disinfected according to infection prevention and control guidelines
	The hospital has infrastructures and protocols for waste management, including clinical and biological waste management
	There are records of all people who enter the COVID-19 patient room; the list includes hospital staff and guests and their contact details (home address, email, and mobile number)
	There are areas and guidelines for handling corpses with COVID-19, including guidelines for the burial of bodies safely

### 3.3. Data analysis

Table 2 shows the assessment criteria to assess each component criteria.

**Table 2:** Assessment criteria.

Criteria	Score
Not available	0
Partially functional	0.5
Fully functional	1

- Not Available: there is no plan, or there is a plan but it has not started yet;
- Partially Functional: planning is already available but not yet comprehensive; or
- Fully Functional: effective and efficient planning, comply with applicable standards.

Analysis methods applied in this study was based on the Rapid hospital readiness checklist for COVID-19 to determine the hospital disaster preparedness (15). Each checklist subcomponent in the checklist is rated according to the following assessment criteria:

Component Score=∑Subcomponent Score

The Component Score is then divided by the number of sub-components in each component as in the following equation:

$$\mathrm{Achievement Percentage}=\frac{\mathrm{Subcomponent Score}}{\sum \mathrm{SubComponent}}$$

The Achievement Percentage was then analyzed according to the level of hospital preparedness in dealing with the COVID-19 pandemic using WHO scoring classification, which is classified into three levels as shown in Table 3 (11):

- Not ready: if the percentage of fulfillment is less than 50%;
- Moderate/Medium Readiness Level: if the percentage of fulfillment is 50–79%; or
- Adequate/High Readiness Level: if the percentage of fulfillment is more than 80%.

**Table 3:** Achievement percentage categories.

Categories	Score
Not ready	≤50%
Partially functional	50–79%
Fully functional	≥80%

### 3.4. Ethical Considerations

This study was approved under the ethical clearance Number Ket- 552/UN2.F10.D11/PPM.00.02/2023 issued by the ethics committees of the Research and Community Engagement, Faculty of Public Health, Universitas Indonesia.

## 4. Results

**Figure 1:**
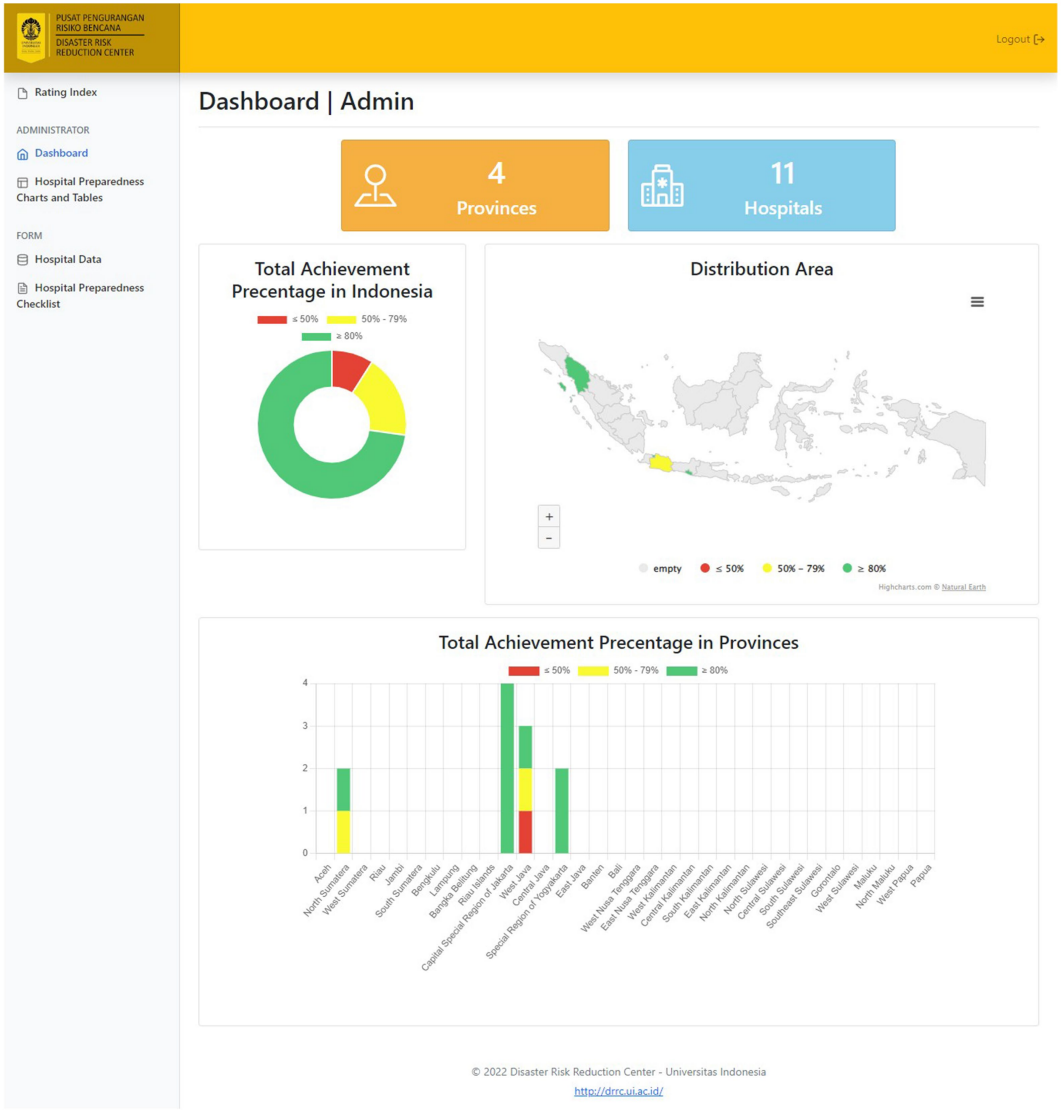
Dashboard of COVID-19 hospital preparedness information system.

**Figure 2:**
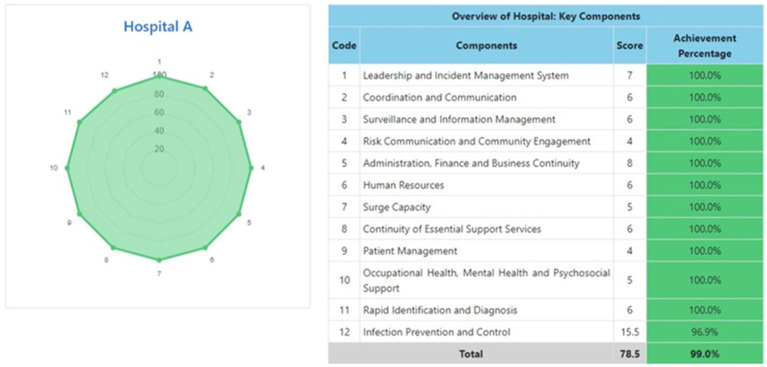
Radar chart and table of COVID-19 hospital preparedness at hospital A in capital Special Region of Jakarta.

**Figure 3:**
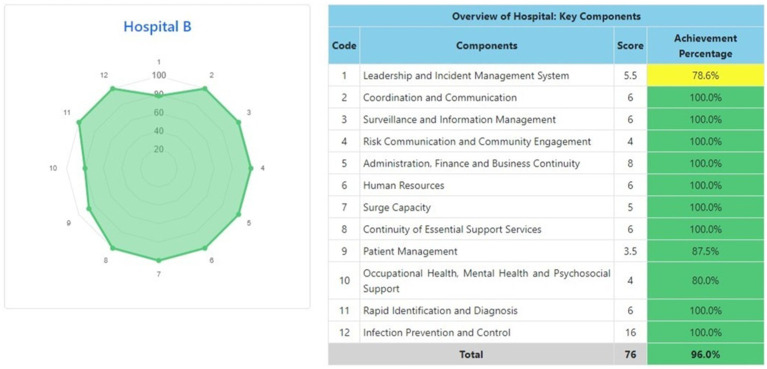
Radar chart and table of COVID-19 hospital preparedness at hospital B in capital Special Region of Jakarta.

**Figure 4:**
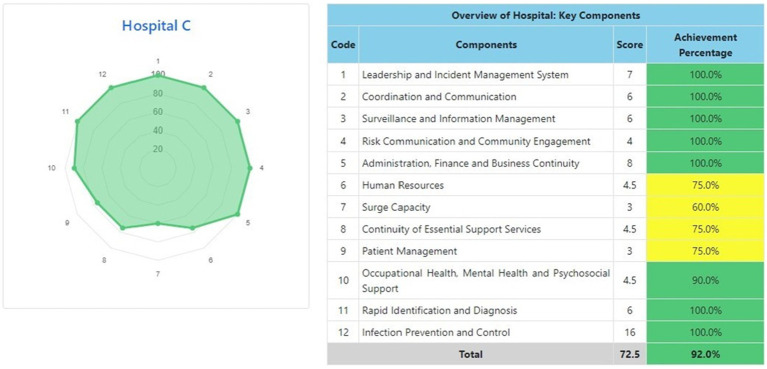
Radar chart and table of COVID-19 hospital preparedness at hospital C in capital Special Region of Jakarta.

**Figure 5:**
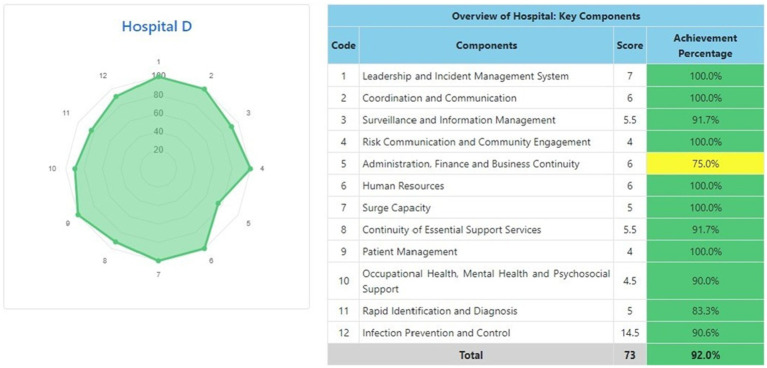
Radar chart and table of COVID-19 hospital preparedness at hospital D in capital Special Region of Jakarta.

**Figure 6:**
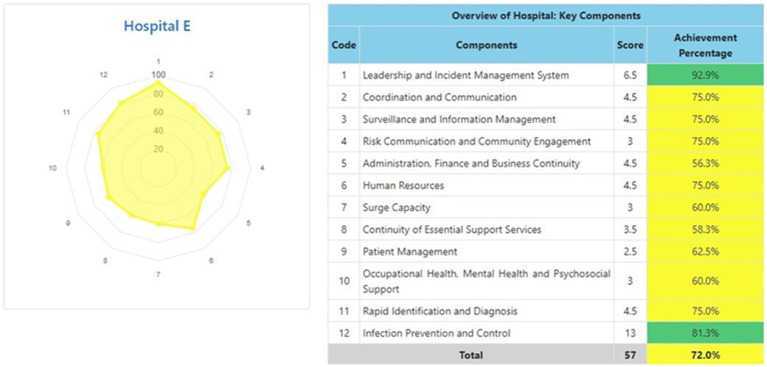
Radar chart and table of COVID-19 hospital preparedness at hospital E in West Java.

**Figure 7:**
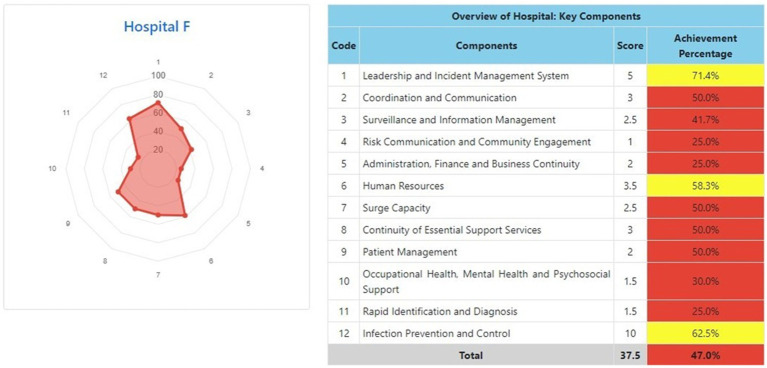
Radar chart and table of COVID-19 hospital preparedness at hospital F in West Java.

**Figure 8:**
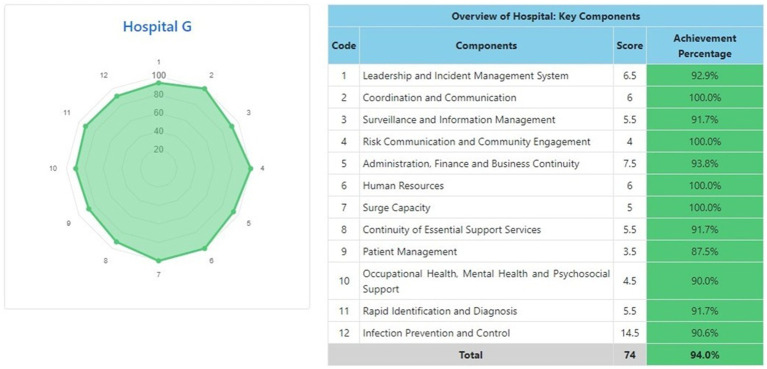
Radar chart and table of COVID-19 hospital preparedness at hospital G in West Java.

**Figure 9:**
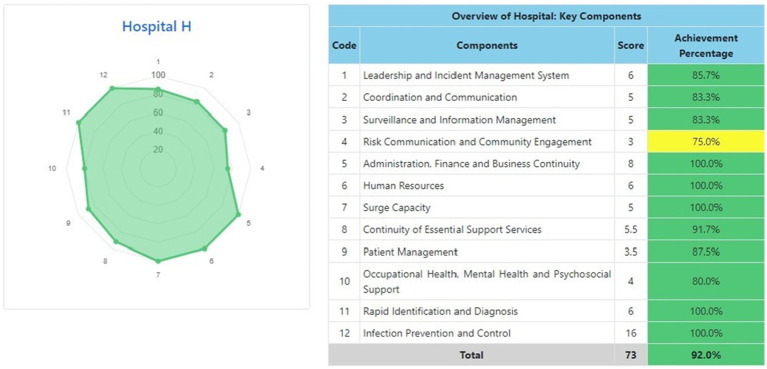
Radar chart and table of COVID-19 hospital preparedness at hospital H in Special Region of Yogyakarta.

**Figure 10:**
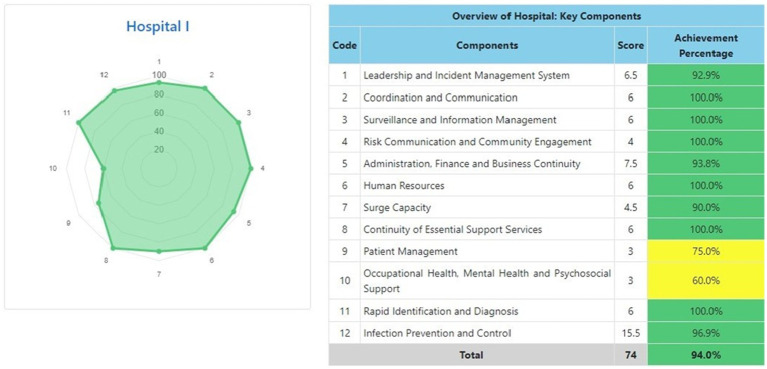
Radar chart and table of COVID-19 hospital preparedness at hospital I in Special Region of Yogyakarta.

**Figure 11:**
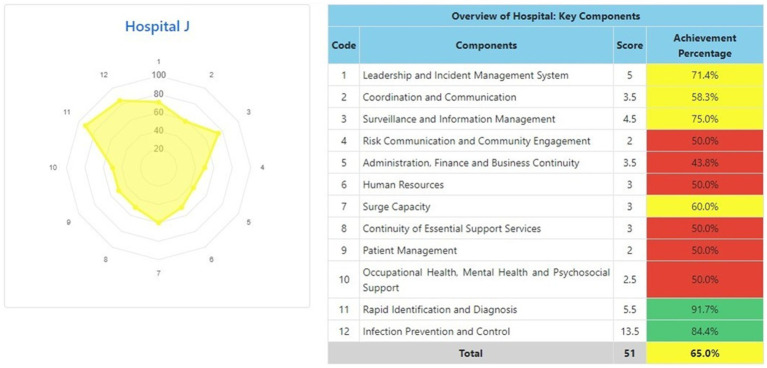
Radar chart and table of COVID-19 hospital preparedness at hospital J in North Sumatra.

**Figure 12:**
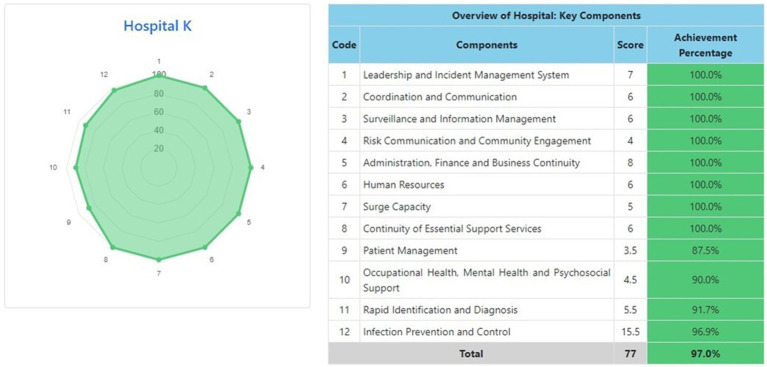
Radar chart and table of COVID-19 hospital preparedness at hospital K in North Sumatra.

After respondents filled out the form in the COVID-19 Hospital Preparedness information system, the entire data are displayed on the information system dashboard as follows:

The information system dashboard (Figure 1) displays the number of provinces and hospitals that have filled out the form, the distribution areas of Hospital Preparedness for COVID-19 assessment in Indonesia, and the number of hospitals’ achievement percentage categories [Not Ready (≤50%), Moderate or Medium Readiness Level (50–79%), and Adequate or High Readiness Level (≥80%)] in each province and for the whole country. From the COVID-19 Hospital Preparedness assessment, Radar Chart, Score, and Percent Achieved can be shown in the COVID-19 Hospital Preparedness information system for 11 hospitals in four provinces [Capital Special Region of Jakarta (4 Hospitals) as shown in Figures 2–5, West Java (3 Hospitals) as shown in Figures 6–8, Special Region of Yogyakarta (2 Hospitals) as shown in Figures 9, 10, and North Sumatra (2 Hospitals) as shown in Figures 11, 12] as follows:

### 4.1. Achievement percentage

Table 4 shows that the majority of hospitals under investigation have achievement percentage in Adequate or High Readiness Level (≥80%). One hospital in West Java (Hospital E) and another hospital in North Sumatra (Hospital J) have Moderate or Medium Readiness Level (50–79%), while one hospital in West Java (Hospital F) is at Not Ready level (≤50%).

**Table 4:** Comparison of achievement percentage of COVID-19 hospital preparedness at 11 hospitals and classified based on the provinces: capital special region of Jakarta (Hospital A, Hospital B, Hospital C, and Hospital D); West Java (Hospital E, Hospital F, and Hospital G); Special Region of Yogyakarta (Hospital H and Hospital I); and North Sumatra (Hospital J and Hospital K).

No	Components	Hospital achievement percentage										
		Capital Special Region of Jakarta				West Java			Special Region of Yogyakarta		North Sumatra	
		A	B	C	D	E	F	G	H	I	J	K
1	Leadership and incident management system	100%	97%	100%	100%	93%	71%	93%	86%	93%	71%	93%
2	Coordination and communication	100%	100%	100%	100%	75%	50%	100%	83%	100%	58%	100%
3	Surveillance and information management	100%	100%	92%	92%	75%	42%	92%	83%	100%	75%	100%
4	Risk communication and community engagement	100%	100%	100%	100%	75%	25%	100%	75%	100%	50%	100%
5	Administration, finance, and business continuity	100%	100%	100%	75%	56%	25%	94%	100%	94%	44%	94%
6	Human resources	100%	100%	75%	100%	75%	58%	100%	100%	100%	50%	100%
7	Surge capacity	100%	100%	60%	100%	60%	50%	100%	100%	90%	40%	100%
8	Continuity of essential support services	100%	100%	75%	92%	58%	50%	92%	92%	100%	50%	100%
9	Patient management	100%	88%	75%	100%	63%	50%	88%	88%	75%	50%	88%
10	Occupational health, mental health, and psychosocial support	100%	80%	90%	90%	60%	30%	90%	80%	60%	50%	90%
11	Rapid identification and diagnosis	100%	100%	100%	83%	75%	25%	92%	100%	100%	92%	83%
12	Infection prevention and control	97%	100%	100%	91%	81%	63%	91%	100%	97%	84%	100%
Total		99.8%	97%	89%	94%	71%	45%	94%	91%	92%	60%	96%

### 4.2. Average achievement percentage

Table 5 indicates that four hospitals in Central Special Region of Jakarta and two hospitals in Special Region of Yogyakarta have average achievement percentage at Adequate or High Readiness Level (≥80%). Meanwhile, the other two provinces (West Java (three hospitals) and North Sumatra (two hospitals)) have average achievement percentage in Moderate or Medium Readiness Level (50–79%).

**Table 5:** Comparison of the average achievement percentage of COVID-19 hospital preparedness from 11 hospitals in four provinces in Indonesia: Capital Special Region of Jakarta, West Java, Special Region of Yogyakarta, and North Sumatra.

No	Components	Average achievement percentage			
		Central Special Region of Jakarta	West Java	Special Region of Yogyakarta	North Sumatra
1	Leadership and incident management system	99%	86%	90%	82%
2	Coordination and communication	100%	75%	92%	79%
3	Surveillance and information management	96%	70%	92%	88%
4	Risk communication and community engagement	100%	67%	88%	75%
5	Administration, finance, and business continuity	94%	58%	97%	69%
6	Human resources	94%	78%	100%	75%
7	Surge capacity	90%	70%	95%	70%
8	Continuity of essential support services	92%	67%	96%	75%
9	Patient Management	91%	67%	82%	69%
10	Occupational health, mental health, and psychosocial support	90%	60%	70%	70%
11	Rapid identification and diagnosis	96%	64%	100%	88%
12	Infection prevention and control	97%	78%	99%	92%
Total		95%	70%	92%	78%

## 5. Discussion

### 5.1. Average achievement percentage

Incident Management System (IMS) is a standardized structure and approach adopted by WHO to administer the response to public health events and emergencies and to ensure that the organization follows best practices in emergency management (18). Good leadership and a well-functioning hospital incident management system team are fundamental for adequately administering emergency operations. Because many hospitals and other healthcare facilities already have critical management and emergency readiness plans, WHO advises adapting these plans to the core requirements for responding to the COVID-19 outbreak and maintaining the hospital’s essential routine of health services (15, 18).

This research found that the leadership and incident management system is at Adequate or High Readiness Level (≥80%) with only two hospitals at Moderate or Medium Readiness Level (50–79%), which results in a good enough. Cooperation between hospital staff and all stakeholders will create a well-functioning hospital incident management system.

This research also proposed findings that efforts to reduce the risk of morbidity and mortality from COVID-19 depend on more than just the availability of health services such as hospitals. However, the social determinants of health also influence the outcome of COVID-19, such as poverty, low-paid workers, crowded housing, and homeless people. Homeless people are at high risk of contracting the disease because they live in dense residential areas, while poor people have limited access to screening and testing. Thus, in addition to health services, the social determinants of health must be prioritized in handling a pandemic. Various improvements that can be made include financial support for low-income families, ease of access to screening and testing, and increasing access to health services for low-income people to reduce the risk of morbidity and death from COVID-19.

### 5.2. Coordination and communication

Appropriate communication and timely coordination are essential to ensure that data inform risk analyses and decision-making; and that there is effective collaboration, cooperation, and credence among all hospital staff and stakeholders. This component includes communication and coordination within the hospital and adequate links with local and national stakeholders, including communities and primary healthcare services (7, 12, 15, 19). Some criteria assessed in this component are divided into internal communication (within the hospital) and external communication and coordination (with other stakeholders).

In this research, the coordination and communication aspects are mostly at Adequate or High Readiness Level (≥80%). Nevertheless, three hospitals are at Moderate or Medium Readiness Level (50–79%) and Not Ready level (≤50%). Therefore, cooperation between the hospital’s countermeasures team or task force and all stakeholders is needed to increase the hospital’s internal and external communication and coordination readiness.

### 5.3. Surveillance and information management

Global surveillance for COVID-19 is a fundamental activity needed to monitor and control pandemic outbreaks, especially in hospitals and long-term healthcare facilities. Hospital information management is equipped with surveillance aspects that are very important to increase public awareness about surveillance, emergency risks associated with public health, and the steps needed to minimize and respond to emergencies (15, 20).

Overall, this study’s surveillance and information management is at Adequate or High Readiness Level (≥80%). That means that the readiness of hospitals in the sample locations have met the WHO preparedness criteria. However, some hospitals were at Moderate or Moderate Readiness Level (50–79%) and Not Ready level (≤50%). Specifically, the following components need improvements: making SOP for collecting, confirming, and validating COVID-19 data; staff to collect, analyze and disseminate data related to COVID-19; and documentation, storage, and backup systems for COVID-19 information.

### 5.4. Risk communication and community engagement

Ensuring effective risk communication and community engagement helps to limit or stop the spread of issues regarding a pandemic outbreak. It can convey precise and clear information concerning COVID-19 (15, 21). Again, although the majority of the hospitals have Adequate or High Readiness Level (≥80%), in this aspect is at some hospitals are at Moderate or Medium Readiness Level (50–79%) and even Not Ready level (≤50%). That shows that a few hospitals’ risk communication and community engagement sub-components are either partially functional or unavailable. Therefore, hospital staff and stakeholders should focus on improving risk communication protocols for infection prevention and control for all staff, patients, guests, community members, and other stakeholders; and COVID-19 risk communication should be developed and updated based on developments in the situation and evidence-based technical guidelines.

### 5.5. Administration, finance, and business continuity

Hospital or facility administration and finance activities are essential to support systems for preventing, preparing, and responding to emergencies during the COVID-19 pandemic (15, 22). Similar to the previous dimensions, hospitals’ administration, finance, and business continuity comprehensively are generally at the Adequate or High Readiness Level (≥80%). However, some hospitals are at Moderate or Medium Readiness Level (50–79%) and Not Ready level (≤50%). This dimension needs to be enhanced by creating a strategy to prevent staff burnout due to the COVID-19 workload and ensure the continuity of healthcare; creating methods for assessing and identifying the expansion of inpatient, outpatient, and Intensive Care Unit (ICU) areas if COVID-19 cases increases; creating COVID-19 plans to refer or outsource non-critical services to appropriate alternative health facilities; and develop hospital business continuity plans to deal with the COVID-19 pandemic.

### 5.6. Human resources

Humans are the most critical resource for preventing, preparing for, responding to, and recovering from a disease outbreak. It is essential to review staffing necessities to establish that hospitals and other healthcare facilities have the appropriate staff and the ability to deliver quality care to respond to the demands caused by the outbreak (15, 23). Resources provision for the COVID-19 control response must support the implementation of medical, laboratory, and other component responses. The provision of these resources needs to be carried out by the Central Government in collaboration with the Regional Government (48).

This research reveals that some hospitals must improve several aspects, such as having adequate staff for preparedness and response to the spike potential of COVID-19 cases, and procedures for monitoring occupational health hazards are available to ensure the safety of hospital staff to mitigate the COVID-19 risk.

### 5.7. Surge capacity

Responses to this component aim to enable hospitals to develop their ability to manage a sudden or rapidly progressive surge in demand for emergency services. COVID-19 may cause a rapid and continued increase in demand. The essential services and supplies needed to resolve the COVID-19 risk include essential healthcare, equipment, and supplies necessary to maintain high-quality healthcare, especially for patients with severe COVID-19 cases. Furthermore, an increased workload should be anticipated (15, 24, 25).

In this research, some hospitals should have plans for capacity enhancement and rapid additions of COVID-19 cases; procedures for COVID-19 management to improve supply chains for essential medicines and diagnostics; and agreements and Memorandum of Understanding (MoU) with the Ministry of Health or other institution to purchase equipment needed for capacity enhancement.

### 5.8. Continuity of essential support services

Besides the COVID-19 pandemic outbreak, which evolves and needs rapid scale-up of emergency preparedness and operational readiness, there are also existing requirements for essential medical and surgical care that requires a hospital’s attention (e.g., emergency medical and surgical services). Therefore, hospitals must consider the best practice to continue and sustain the continuity of their health services safely (e.g., in terms of supplies, logistics, and pharmacy services) while addressing COVID-19 case management needs (15, 26–28).

In this research, certain hospitals should identify and prioritize essential support services that must be available at all times and situations with sufficient resources and backups to ensure the continuity of these services; maintenance, supplies, and inventory systems are available; security system has identified potential health and safety hazards and mitigation plans for security risks; has the expansion plan for clinical management and the waste management of the hospital integrated with the water, sanitation, and hygiene (WASH) system; and information management system to monitor routine essential health service usage that is not related to COVID-19 through predetermined indicators.

### 5.9. Patient management

Patient management consists of admission or referral, triage, diagnosis, treatment, patient flow and tracking; discharge and follow-up; support services management, pharmacy services, logistics, and supply functions. The purposes are to ensure that the hospital’s patient management system remains safe, effective, and efficient and that the hospital can manage safe and effective patient management when normal circumstances and COVID-19 pandemic demands increase the hospital’s resources and capacities. When dealing with a new infectious disease outbreak, hospitals should ensure they have space for triage and isolating COVID-19 suspected, possible, and confirmed cases (15, 29–35, 49).

In this research, some hospitals should update protocols to provide essential care services for COVID-19 patients based on WHO guidelines; have procedures and provisions for receiving patients and transferring them to hospital isolation rooms or other diagnostic and therapeutic support services; have protocols for actions that have not been clinically tested, are still trial or unregistered emergency measures, ethically approved clinical trials or Monitored Emergency Use of Unregistered Interventions (MEURI framework); and hospital staff have to implement prevention protocol, infection control, transport services before and after referral, including patients transferred from homecare services.

### 5.10. Occupational health, mental health, and psychosocial support

Occupational health, mental health, and psychosocial support services are necessary to decrease the detrimental psychological and social impacts of COVID-19 on hospital patients, staff, families, and communities. WHO has published guidelines regarding assessing and managing risks to healthcare staff. Additionally, several publications review the mental health and psychosocial issues associated with the pandemic (15, 36–39).

Certain hospitals, particularly outside the Capital Special Region of Jakarta, should protect, train, and equip their staff with Personal Protective Equipment (PPE) in providing medical services to patients with suspected, probable, or confirmed cases of COVID-19; have the policy and capacity to manage occupational safety and health and prevent infection control from protecting hospital staff; have psychosocial and mental health support for hospital staff, family, and patients; and all hospital staff have been trained in essential Occupational Safety and Health (OSH) and psychological first aid.

### 5.11. Rapid identification and diagnosis

The rapid identification and diagnosis laboratory of COVID-19 cases will ensure a logical and effective chain of events during case management. In principle, the triage process identifies patients who need treatment and immediate medical intervention or may need to access certain health facilities based on the patient’s clinical condition (14).

The result indicates that some hospitals should train their staff regarding rapid identification accurately and timely screening of suspected COVID-19 cases; have communication and monitoring systems for vigilance and reporting of suspected COVID-19 cases in all hospital areas; have triage procedures in the emergency unit, focusing on rapid identification, isolation, and testing of patients with acute respiratory infection symptoms; and train hospital staff concerning standard procedures for taking samples and sending them to referral laboratories. Laboratory services must be provided to support the hospital’s preparedness, operational readiness, and response activities, such as surveillance, infection prevention and control (IPC), and patient management; all of these must be accomplished promptly and efficiently (15, 31, 40).

### 5.12. Infection prevention and control

Based on the Regulation of the Minister of Health of the Republic of Indonesia number 27 of 2017 concerning Infection Prevention and Control, Infection Prevention and Control (IPC) is an effort to prevent and minimize the occurrence of infections in patients, staff, visitors, and the community around healthcare facilities (48). IPC is essential to minimize the COVID-19 transmission risk to hospital staff, other patients, close contacts, and visitors. Prevent or breaking the chain of COVID-19 infection transmission in healthcare facilities can be achieved by applying the principles of COVID-19 prevention and transmission control risk, which consists of isolation precautions (standard and transmission precautions), administrative controls, education and training; PPI in pre-referral health facilities; and IPC to burial corpses (15, 50).

In this research, only one hospital is at Not Ready level (≤50%), while the rest are at Adequate or High Readiness Level (≥80%). Cooperation between hospital staff, the countermeasure task force team, and all stakeholders is key to ensuring hospital infection prevention and control.

This study has several limitations. Firstly, the sample size of selected hospital was quite small (only 11 hospitals), which delivered from four provinces in Indonesia. Further study from another countries is required to be conducted in order to assess the hospital preparedness situations. Secondly, since the study focus on the hospital preparedness for COVID-19 situation, the selected hospital was not considered the characteristic of employees. It was based on the hospital that is the referral for Covid-19 patients. Thirdly, the data collection was very limited and done through online focus group discussion and interviews, therefore it is strongly recommended for further study to conduct a direct survey. Fourthly, the instrument used for study is based on WHO checklist which proposed in COVID-19 situation, which the first study conducted in Indonesia. The study in these areas is strongly recommended to examine especially in pre-COVID-19 situation and post COVID-19 as well as Hospital preparedness for preventing pandemic in future. Lastly, further statistical analysis such multivariate analysis method should be considered to identify the most significant variables that affect Hospital preparedness.

## 6. Conclusion

Hospital preparedness for COVID-19 needs to become a concern for assessment and evaluation. Limited research, especially in Indonesia, related to hospital preparedness for COVID-19 makes this research necessary. Based on the analysis, Central Special Region of Jakarta (Hospital A and Hospital B), West Java (Hospital G), and North Sumatra (Hospital K) with overall scores at Adequate or High Readiness Level (≥80%) for 12 components of Rapid hospital readiness checklist for COVID-19. Despite some elements are still found to have a poor level of preparedness, especially Hospital E in West Java was at Moderate or Medium Readiness Level (50–79%) and Hospital F in West Java was at Not Ready level (≤50%) in almost all aspects such as coordination and communication; surveillance and information management; risk communication and community engagement; administration, finance and business continuity; human resources; surge capacity; continuity of essential support services; patient management; occupational health, mental health, and psychosocial support; and rapid identification and diagnosis.

The assessment results also demonstrate that the hospital preparedness in the Central Special Region of Jakarta and Special Region of Yogyakarta tend to be better if compared with the other two provinces (West Java and North Sumatra) in some aspects: coordination and communication; risk communication and community engagement; administration, finance and business continuity; human resources; surge capacity; continuity of essential support services; and patient management.

The results of this research can be used to inform hospital staff, countermeasures team or task force, and relevant stakeholders regarding COVID-19 hospital awareness and preparedness then appropriate policies and practice guidelines can be implemented to improve their capabilities of facing this pandemic outbreak and other future pandemic-prone diseases. This study has limitations regarding the small number of provinces and hospitals which have filled out forms in COVID-19 Hospital Preparedness information system. Further studies are needed to describe the overall hospital preparedness for COVID-19 in Indonesia. It is also envisioned that the dashboard can be expanded over time by assessing hospital preparedness against various disaster events.

## Data availability statement

The data analyzed in this study is subject to the following licenses/restrictions: The datasets utilized and/or analyzed during the present study are available on reasonable request from the corresponding author. Requests to access these datasets should be directed to fatma@ui.ac.id.

## Author contributions

FL and AK: conceptualization, methodology, and funding acquisition. AP: software, validation, and visualization. OW, S, HE-M, and DL: formal analysis. FA: investigation and project administration. DL: resources. AYH: data curation. FL, AK, OW, HE-M, and S: writing—original draft preparation. RS: writing—review and editing. FL: supervision. All authors contributed to the article and approved the submitted version.

## Funding

This study is funded by Directorate of Research and Development, Universitas Indonesia, Under Hibah PUTI Q1 (Grant No. NKB-462/UN2.RST/HKP.05.00/2022).

## Conflict of interest

The authors declare that the research was conducted in the absence of any commercial or financial relationships that could be construed as a potential conflict of interest.

## Publisher’s note

All claims expressed in this article are solely those of the authors and do not necessarily represent those of their affiliated organizations, or those of the publisher, the editors and the reviewers. Any product that may be evaluated in this article, or claim that may be made by its manufacturer, is not guaranteed or endorsed by the publisher.
